# Preclinical Validation of [^177^Lu]Lu-AKIR001, a CD44v6-Targeted Radiotherapeutic Entering First-in-Human Trials

**DOI:** 10.2967/jnumed.125.270782

**Published:** 2026-02

**Authors:** Anja C.L. Mortensen, Tabassom Mohajershojai, Amanda Gustafsson, Hanna Berglund, Ram Kumar Selvaraju, Camilla Hofström, Helena Persson, Mats Ohlin, Thuy A. Tran, Anton Forsberg Morén, Piotr Ochniewicz, Jan Zedenius, Peter Bernhardt, Fredrik Y. Frejd, Marika Nestor

**Affiliations:** 1 Department of Molecular Medicine and Surgery, Karolinska Institutet, Stockholm, Sweden;; 2 Department of Immunology, Genetics, and Pathology, Science for Life Laboratories, Uppsala University, Uppsala, Sweden;; 3 Department of Medicine, Karolinska Institutet, Stockholm, Sweden;; 4 Preclinical PET-MRI Platform, Department of Medicinal Chemistry, Uppsala University, Uppsala, Sweden;; 5 Drug Discovery and Development, Science for Life Laboratories and Royal Institute of Technology, Stockholm, Sweden;; 6 Drug Discovery and Development, Science for Life Laboratories and Department of Immunotechnology, Lund University, Lund, Sweden;; 7 Department of Oncology and Pathology, Karolinska Institutet, Stockholm, Sweden;; 8 Department of Clinical Neuroscience, Karolinska Institutet, Stockholm, Sweden;; 9 Department of Breast, Endocrine Tumors, and Sarcoma, Karolinska University Hospital, Stockholm, Sweden;; 10 Department of Medical Radiation Sciences, Institute of Clinical Sciences, Sahlgrenska Academy at University of Gothenburg, Gothenburg, Sweden; and; 11 Department of Medical Physics and Biomedical Engineering, Sahlgrenska University Hospital, Gothenburg, Sweden

**Keywords:** CD44v6, radioantibody–drug conjugates, rADCs, RPT, translational oncology

## Abstract

Targeted radionuclide therapy is an emerging potent therapeutic strategy in oncology. The cell surface antigen CD44v6 is a potential pan-cancer target for radionuclide therapy. This study aimed to evaluate the therapeutic efficacy, biodistribution, dosimetry, and safety profile of AKIR001, an antibody targeting CD44v6 labeled with ^177^Lu. **Methods:** The biodistribution and preclinical dosimetry of [^177^Lu]Lu-AKIR001 were calculated in the highly CD44v6-expressing A431 murine xenograft model, with subsequent extrapolation to predict human dosimetry. Therapeutic efficacy was evaluated across 3 xenograft models, 2 with high and 1 with moderate levels of CD44v6, using multiple dosing levels, fractionation regimens, and combinations with cisplatin. Preclinical toxicology was evaluated in a cross-reactive rabbit model and complemented by a PET imaging study using ^68^Ga-labeled AKIR001 in a cynomolgus macaque. **Results:** Biodistribution studies confirmed the high and selective tumor uptake of [^177^Lu]Lu-AKIR001, resulting in favorable dosimetry predictions for clinical application. Therapeutic evaluations demonstrated significant dose-dependent efficacy in all tested xenograft models, with fractionated dosing (2 doses) resulting in complete tumor regression in 80% of the animals in a radioresistant xenograft model. Biodistribution in rabbits demonstrated low uptake in normal tissues, and a good-laboratory-practice study using an excessive dose of AKIR001 was well tolerated, with no signs of adverse effects. PET imaging in a cynomolgus macaque corroborated these findings. **Conclusion:** Collectively, these data strongly support the therapeutic efficacy, safety, and dosimetry of [^177^Lu]Lu-AKIR001, justifying its advancement into clinical trials. A phase 1 clinical trial of [^177^Lu]Lu-AKIR001for CD44v6-positive solid cancers (NCT06639191) is currently recruiting patients.

## Visual Abstract

**Figure:**
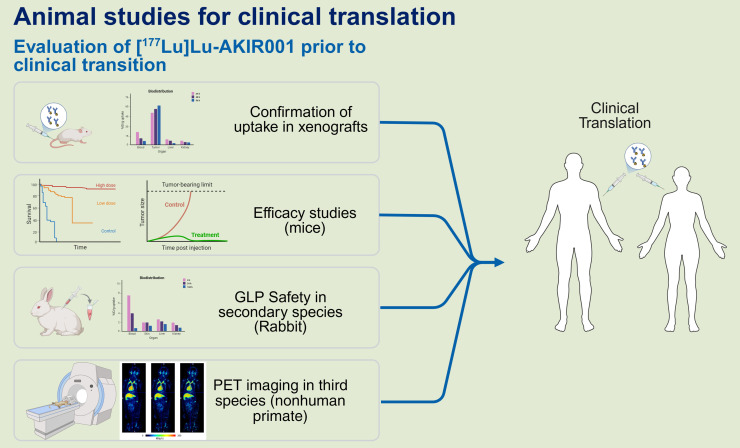

Radiopharmaceutical therapy (RPT) delivers ionizing radiation directly to tumors by attaching a radionuclide to a carrier molecule that selectively binds tumor-specific structures (1). Over the past decade, RPT has achieved notable clinical success, exemplified by the approvals of [^177^Lu]Lu-DOTATATE and [^177^Lu]Lu-PSMA-617. Despite these advances, their clinical application is limited by the restricted expression of their respective targets—the somatostatin receptor and prostate-specific membrane antigen (PSMA) (1).

Currently, peptides and small molecules dominate as targeting agents in RPT, but many tumor antigens lack the binding pockets necessary for developing highly specific low-molecular-weight agents with sufficient affinity. This limitation has prompted efforts to diversify targeting strategies to broaden the range of accessible tumor antigens. For RPT to be effective, the radiolabeled agent must remain bound in the tumor long enough to deliver a therapeutic radiation dose while minimizing exposure to normal tissues. Achieving this requires targeting agents with exceptionally high affinity and specificity, which can be challenging for certain tumor antigens. Antibodies, with their inherent high affinity and specificity, present a promising alternative. Antibody-based RPT, known as radioantibody–drug conjugates, is gaining traction, supported by recent advances in antibody engineering and radiopharmaceutical development (2). However, because of their large size and prolonged circulation time, it is essential to select targets that are highly and selectively overexpressed in tumors to optimize efficacy and minimize off-target toxicity.

To expand RPT’s applicability, research has focused on identifying tumor-associated targets with broad expression profiles. Fibroblast activation protein has been proposed as a pan-cancer target, but its stromal localization and expression in noncancerous diseases raise concerns about toxicity in nontumor tissues (3). As an alternative, CD44v6, a splice variant of the cell surface glycoprotein CD44, has emerged as a promising target (4). CD44v6 is highly overexpressed in a plethora of epithelium-derived malignancies, and expression in normal tissues is largely restricted to subsets of epithelial tissues. Expression levels vary within malignancies, with skin and head-and-neck squamous cell carcinomas demonstrating the highest level of overexpression (>90%), whereas lung and breast cancers demonstrate a wide interval of expression levels (0%–95% and 24%–88%, respectively) (5). Expression of CD44v6 correlates with tumor aggressiveness and metastatic potential and is generally retained in metastatic lesions, making it a compelling target for therapy (5). The association of CD44v6 with poor prognosis further highlights the unmet medical need for effective treatments in CD44v6-positive cancers.

Previous clinical studies of CD44v6-targeted RPT using [^186^Re]Re-BIWA-4 (bivatuzumab) demonstrated promising efficacy and a favorable safety profile in patients with head and neck squamous cell carcinoma, although subsequent development as an antibody–drug conjugate was halted because of skin toxicity (6,7). These findings underscore the potential of CD44v6-targeted RPT, provided that next-generation antibodies can combine high affinity with improved safety. Previous generations of antibody-based therapies have in some cases been limited by Fc-mediated toxicities and on-target/off-tumor effects in normal tissues (8). To address such challenges, next-generation antibodies often incorporate Fc engineering to reduce Fcγ-receptor binding and thereby minimize effector functions while maintaining high affinity and tumor selectivity (8).

We have developed AKIR001, a high-affinity human IgG1 antibody targeting CD44v6 (4,9,10), with the purpose of investigating RPT with a high-affinity binder to CD44v6. AKIR001 incorporates the LALA double mutation (Leu234Ala, Leu235Ala) to reduce normal-tissue uptake dependent on Fcγ-receptor interactions and minimize potential Fc-mediated toxicities, such as antibody-dependent cellular cytotoxicity (ADCC) and complement-dependent cytotoxicity (CDC) (11). Early studies with the parental antibody, [^177^Lu]Lu-UU-40, demonstrated strong efficacy in highly CD44v6-expressing tumors but only moderate effects on tumors with lower expression (4). This prompted an affinity maturation, resulting in the development of AKIR001, which is now advancing toward clinical application for CD44v6-positive cancers (10).

This study evaluated the biodistribution, dosimetry, and therapeutic efficacy of [^177^Lu]Lu-AKIR001 in 3 xenograft models with varying CD44v6 expression; assessed toxicity and biodistribution in rabbits; and used nonhuman primate imaging to further investigate AKIR001’s accumulation in healthy tissues before clinical translation.

## MATERIALS AND METHODS

Full details of the materials and methods can be found in the supplemental materials (available at http://jnm.snmjournals.org) and include the use of human cancer cell lines including ACT-1, BHT-101, A431, SK-BR-3, MCF-7, BxPC-3, and Raji. AKIR001 and its parental antibody UU-40 were produced by SciLifeLab, Absolute Antibody, or Genscript Probio (9). Antibodies were DOTA-conjugated and radiolabeled: ^177^Lu-labeling involved incubating 50–400 µg of antibody with 15–130 MBq of ^177^Lu at 37 °C (≥99% yield); ^111^In-labeling used 50–60 MBq at 42 °C; ^68^Ga-labeling used GalliAd (IRE ELiT) generator eluate and NAP-5 purification; and ^125^I-labeling used Pierce Iodination Tubes (4,10). All labeling yields were assessed by ITLC. CDC and ADCC assays measured cytotoxicity with viability or luminescence readouts. Antigen specificity and density were determined by radiolabeled binding and blocking experiments (4). Tumor models involved female BALB/c *nu/nu* mice inoculated with A431, ACT-1, or BHT-101 cells. Biodistribution studies assessed the distribution, dosimetry, and efficacy of various ^177^Lu-AKIR001 doses, monitoring tumor growth, weight, and hematology for between 40 and 135 d (12). SPECT/CT imaging traced biodistribution, and rabbit and cynomolgus macaque studies evaluated organ uptake and safety.

## RESULTS

### In Vitro Studies

#### CDC and ADCC Not Induced by AKIR001

AKIR001 carries LALA-silencing mutations in its FcγR-binding domain to reduce FcγR and C1q interactions, minimizing tissue interactions and limiting CDC and ADCC risks (13). In vitro studies confirmed that AKIR001 does not induce CDC or ADCC in BxPC-3 cells. The CDC assay demonstrated that, unlike the positive control rituximab, AKIR001 had weak CDC activity (Supplemental Fig. 1A). Similarly, an ADCC assay indicated that AKIR001 lacks ADCC activity, with low cell-killing signals compared with trastuzumab (Herceptin; Roche) (Supplemental Fig. 1B). These findings align with previous SPR assessments of AKIR001’s FcγR-binding properties (10).

#### Specificity of ^177^Lu-AKIR001 to Cells with Varying Target Expression

Cell specificity assays of radiolabeled AKIR001 evaluated cancer cell line specificity. Uptake of radiolabeled AKIR001 was effectively blocked in all positive lines using a 100-fold molar excess of unlabeled antibody (Supplemental Fig. 1C), verifying specificity. No specific uptake occurred in CD44v6-negative MCF-7 cells. ACT-1 and A431 cells revealed the highest uptake (∼3,000,000 antigens per cell), whereas BHT-101 cells had lower uptake (∼250,000 antigens per cell).

### In Vivo Studies

#### Biodistribution Studies in Mice

**FIGURE 1.**
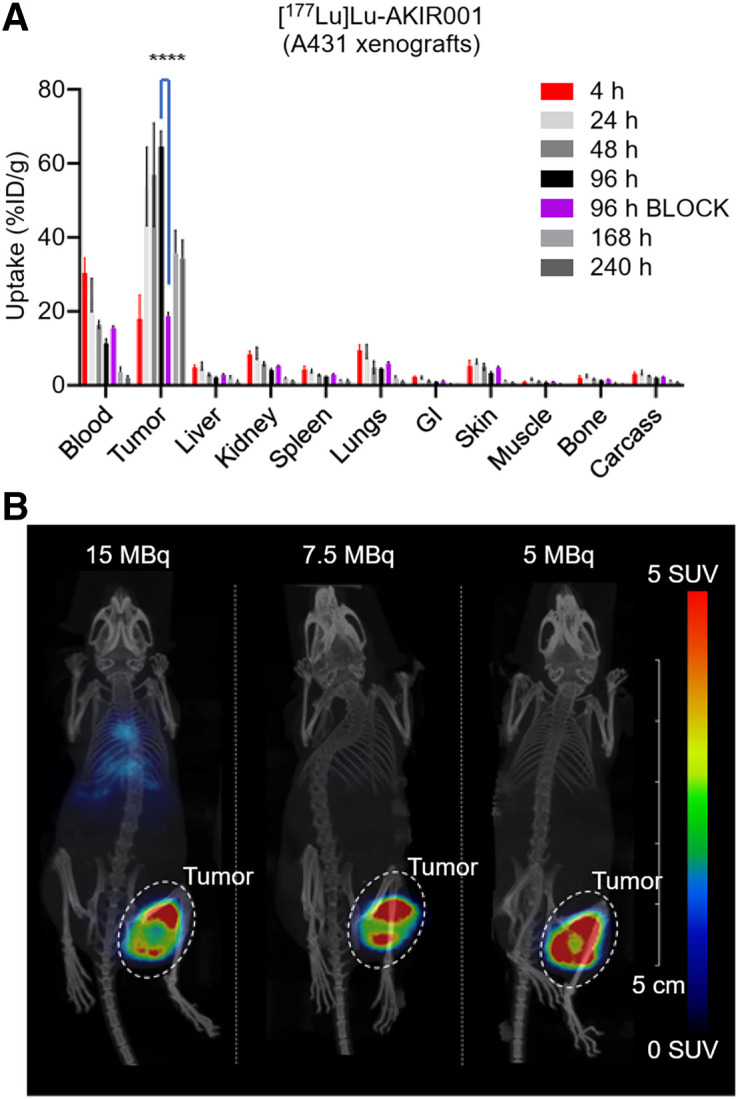
Biodistribution and small-animal SPECT imaging at 3 dose levels of ^177^Lu-AKIR001 in A431 xenografts. (A) Biodistribution of ^177^Lu-AKIR001 (15 µg) in BALB/c nu/nu mice carrying A431 xenografts, terminated at 4, 24, 48, 96, 168, and 240 h after injection. In vivo block using 30-fold molar excess of nonradiolabeled AKIR001 was evaluated at 96 h after injection. Error bars represent SD for *n* ≥ 3 per time point (*N* = 33). ****Student *t* test defined significance between blocked and nonblocked tumor uptake as *P* < 0.0001. (B) SPECT/CT images acquired 6 d after injection with 3 different tracer doses. SPECT images display tracer distribution in SUV units using red–green–blue color scale, whereas corresponding CT images, shown as maximum-intensity projections in gray scale, provide anatomic reference. GI = gastrointestinal tract.

Biodistribution studies were performed in BALB/c nu/nu mice carrying A431 xenografts (high CD44v6 expression). Increasing tumor uptake was observed, peaking at 65 %ID/g at 96 h after injection (Fig. 1A). Blocking with an excess of unlabeled AKIR001 reduced uptake to 19 %ID/g (*P* < 0.0001), confirming specificity. The uptake in all normal organs peaked at the earliest time point (4 h), and tumor-to-blood ratios increased over time, culminating at 17.4 at 240 h after injection (Table 1).

**TABLE 1.** Tumor-to-Organ Ratios of [^177^Lu]Lu-AKIR001 in A431 Xenografts

**Organ**	**4 h**	**24 h**	**48 h**	**96 h**	**96 h block**	**168 h**	**240 h**
Blood	0.6 ± 0.2	2.2 ± 0.5	3.4 ± 0.7	5.7 ± 0.3	1.2 ± 0.1	10.6 ± 3.5	17.4 ± 2.8
Liver	3.8 ± 1.3	10.7 ± 2.8	19.3 ± 3.3	30.6 ± 3.1	6.7 ± 1	26.6 ± 15.2	44.3 ± 18.5
Kidney	2.2 ± 0.8	6.3 ± 1.6	9.7 ± 1.6	16 ± 1.3	3.6 ± 0.2	19.5 ± 1.6	29.5 ± 3.1
Spleen	4.3 ± 1.6	13.9 ± 2.2	20.6 ± 6.4	27 ± 1.7	6.5 ± 0.6	26.1 ± 3.9	30.2 ± 6.5
Lungs	1.9 ± 0.5	6.1 ± 1.6	14.4 ± 10.2	14.3 ± 0.5	3.2 ± 0.4	19.6 ± 4.8	31.5 ± 3.9
GI	7.9 ± 2.8	26.5 ± 6.4	45.1 ± 7.6	67.3 ± 3	17.3 ± 5.3	87.9 ± 7.7	157.1 ± 28.8
Skin	3.8 ± 1.7	8.4 ± 1.8	11.8 ± 4.2	19.5 ± 2	4 ± 0.4	32.8 ± 5.1	46.8 ± 12.9
Muscle	19.7 ± 5.5	34.6 ± 11.4	54.4 ± 17.4	77.5 ± 11	21.8 ± 3.3	110.5 ± 17.7	295.5 ± 111
Bone	8.6 ± 2	21.3 ± 3	33.1 ± 8.9	49.5 ± 5.9	11.7 ± 0.8	73 ± 21.5	154.7 ± 60.9
Carcass	5.9 ± 1.8	16 ± 3.7	21.9 ± 5.4	32.5 ± 2.2	8.3 ± 0.7	35.8 ± 12.3	46.5 ± 11.4

GI = gastrointestinal tract.

Ratios were calculated from biodistribution data presented in Figure 1A.

#### SPECT

Small-animal SPECT confirmed tumor-specific uptake of [^177^Lu]Lu-AKIR001 in A431 xenografts at all tested doses (15, 7.5, and 5 MBq). Imaging at 6 d after injection showed high, specific tumor uptake with excellent contrast and no evidence of off-target accumulation across all doses (Fig. 1B).

### In Vivo Efficacy

Three xenograft models were used to assess the efficacy of [^177^Lu]Lu-AKIR001, each targeting different aspects. The A431 model (high CD44v6, radioresistant) compared single high, single low, and fractionated doses, focusing on tumor response and hematologic toxicity, and was also tested in combination with cisplatin (*cis-*diamminedichloroplatinum [CDDP]) (14). The ACT-1 model (high CD44v6) investigated several negative controls and 2 single doses (high and low). The BHT-101 model (moderate CD44v6) evaluated whether lower-expressing tumors could achieve similar efficacy using a moderate dose.

#### Treatment Effects of Single and Fractionated Doses

**FIGURE 2.**
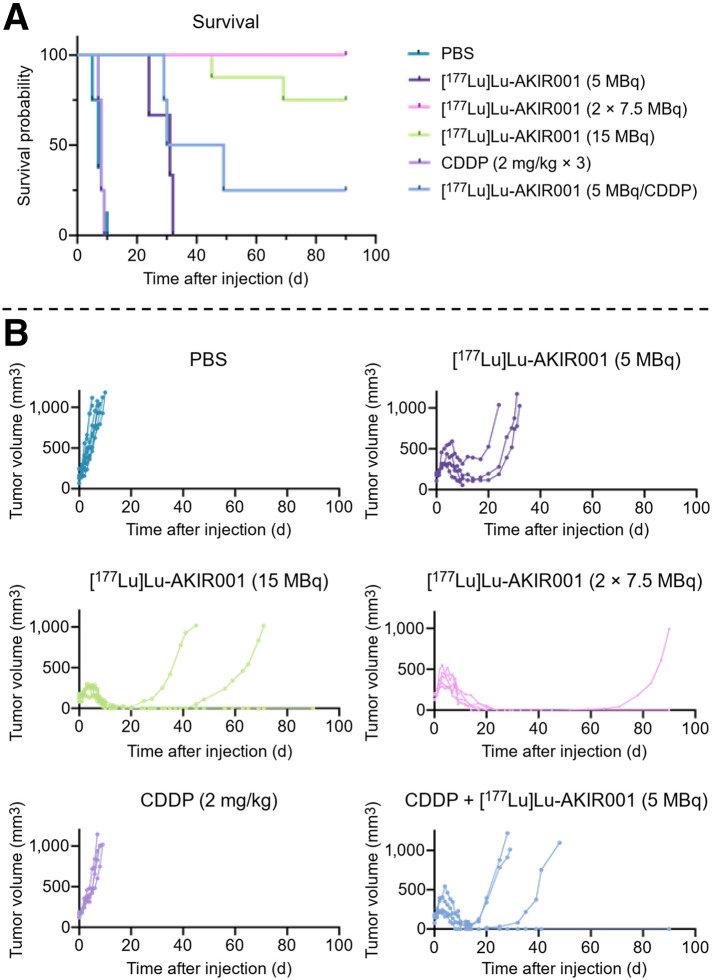
Efficacy of ^177^Lu-AKIR001 in A431 xenografts. (A) Kaplan–Meier survival plot of mice carrying A431 xenografts and injected with placebo (phosphate-buffered saline, *n* = 8) or 15 MBq (*n* = 8), 7.5 MBq (*n* = 5), or 5 MBq (*n* = 3) of [^177^Lu]Lu-AKIR001; with CDDP on 3 consecutive days (*n* = 4) (2 mg/kg) via intraperitoneal injection; or with combination of CDDP and 5 MBq (*n* = 4) of [^177^Lu]Lu-AKIR001. Dose of 7.5 MBq was repeated after 14 d (*N* = 32). (B) Individual tumor growth of A431 xenografts as measured by digital caliper. Tumor-bearing limit was set to 1,000 mm^3^, and study was concluded on day 90. PBS = phosphate-buffered saline.

The A431 xenograft model was used to assess the efficacy of [^177^Lu]Lu-AKIR001 with single high (15 MBq), single low (5 MBq), fractionated (2 × 7.5 MBq, 14 d apart), and placebo (phosphate-buffered saline) groups. Two additional groups also received moderate CDDP (2 mg/kg for 3 consecutive days) as monotherapy or combined with 5 MBq of [^177^Lu]Lu-AKIR001 to evaluate combination effects. Median survival was 7 d for placebo and 8 d for CDDP monotherapy, indicating that CDDP at this dose level did not inhibit tumor growth (Fig. 2A). Median survival was 31 d for the 5-MBq [^177^Lu]Lu-AKIR001 group and 39.5 d for the CDDP combination group. Median survival remained undefined for the high and fractionated groups. All [^177^Lu]Lu-AKIR001–treated animals initially demonstrated tumor regression, with the 15-MBq group regressing comparatively faster. Both the high and the fractionated groups achieved 100% tumor regression (after the second dose was administered), though regrowth occurred in 2 of 8 animals in the high-dose group and 1 of 5 animals in the fractionated group (Fig. 2B). This translated into complete response rates of 75% for the high-dose group and 80% for the fractionated-dose group. In comparison, the CDDP combination group had a 25% complete response rate (1/4 animals). Significant differences in survival probability were observed for [^177^Lu]Lu-AKIR001 (15 MBq [*P* < 0.001], 7.5 MBq [*P* < 0.01], and 5 MBq [*P* < 0.05]) and for 5 MBq plus CDDP compared with the placebo control (phosphate-buffered saline), whereas CDDP monotherapy was not significant. Higher [^177^Lu]Lu-AKIR001 doses (15 and 7.5 MBq) outperformed 5 MBq (*P* < 0.01 and *P* < 0.05, respectively), and both 15 and 7.5 MBq were superior to CDDP (*P* < 0.01 and *P* < 0.05, respectively). Further, the combination therapy performed significantly better than CDDP monotherapy (*P* < 0.05) but did not differ from [^177^Lu]Lu-AKIR001 monotherapy at any of the evaluated doses.

Hematologic toxicity was greater in the high-dose group and fractionated-dose group, but all parameters had recovered by day 57; the low-dose and combination groups showed only mild hematologic effects (Supplemental Fig. 2) and no signs of a decline in weight (Supplemental Fig. 3A).

#### Dose-Dependent and Target-Specific Effect of ^177^Lu-AKIR001

**FIGURE 3.**
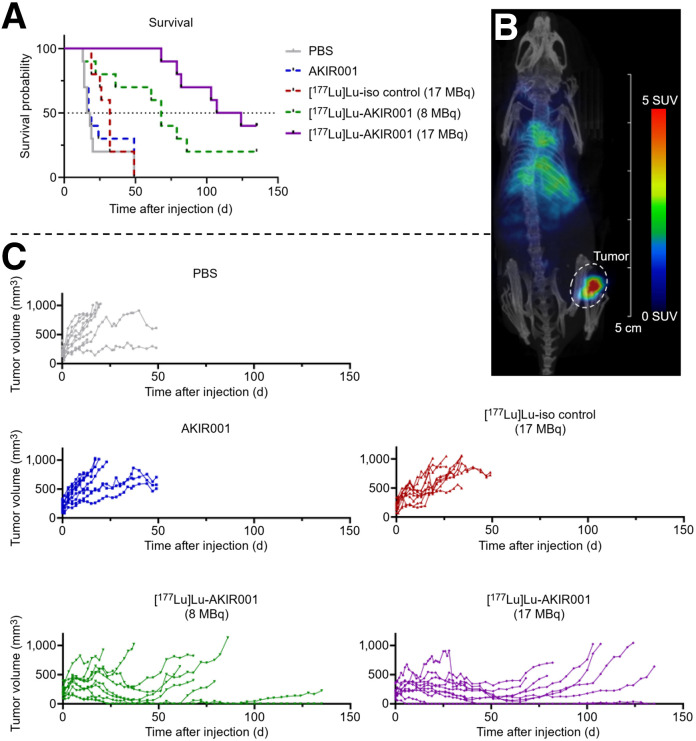
Efficacy studies on ACT-1. (A) Kaplan–Meier survival of animals carrying ACT-1 xenografts (high CD44v6) treated with phosphate-buffered saline, nonradiolabeled AKIR001, [^177^Lu]Lu-iso control (AK-MO176-156, hIgG1 LALA isotype control, 17 MBq), or high dose (17 MBq) or low dose (8 MBq) of [^177^Lu]Lu-AKIR001. (B) Small-animal SPECT of mouse injected with 17 MBq of [^177^Lu]Lu-AKIR001 at 4 d after injection. (C) Individual tumor growth by treatment group. Tumor-bearing limit was 1,000 mm^3^, and study was concluded on day 135 (*N* = 50, *n* = 10 in each group). PBS = phosphate-buffered saline.

The potential effect of the nonradiolabeled antibody and a high dose (17 MBq) of an isotype control antibody, [^177^Lu]Lu-AK-MO176-156, was evaluated using the ACT-1 xenograft model (high CD44v6) in comparison with an equally high dose of [^177^Lu]Lu-AKIR001 (17 MBq) and a lower dose of [^177^Lu]Lu-AKIR001 (8 MBq) (Fig. 3A).

The nonradiolabeled AKIR001 group demonstrated no impact on tumor growth or median survival (18 vs. 17 d for placebo). The [^177^Lu]Lu-AK-MO-176-156 isotype control group modestly extended median survival to 32 d without improving overall survival. The untreated controls (phosphate-buffered saline), nonradiolabeled AKIR001, and ^177^Lu-labeled isotype control antibody did not differ significantly from one another in regard to survival probability, indicating no intrinsic therapeutic effect of the antibody backbone or isotope labeling alone. In contrast, [^177^Lu]Lu-AKIR001 demonstrated a clear dose-dependent effect and significantly improved survival compared with controls: 8 MBq increased median survival 4-fold (68 d, *P* < 0.01), and 17 MBq increased median survival nearly 7-fold (116 d, *P* < 0.0001). Further, [^177^Lu]Lu-AKIR001 treatments significantly improved survival compared with the ^177^Lu-labeled isotype control antibody (*P* < 0.0001 and *P* < 0.05 for 17 and 8 MBq, respectively). After 135 d, 4 high-dose animals, of which 3 demonstrated a complete response (30% complete response rate, 3/10), and 2 animals in the low-dose group, of which 1 animal demonstrated a complete response (10%, 1/10), had survived (Fig. 3C). SPECT confirmed high tumor specificity (Fig. 3B), and no health issues were observed (Supplemental Fig. 3B), though some animals were euthanized early for ethical reasons such as of loss of function in the posterior leg below the tumor.

#### Effects on Xenografts with Moderate CD44v6 Expression

**FIGURE 4.**
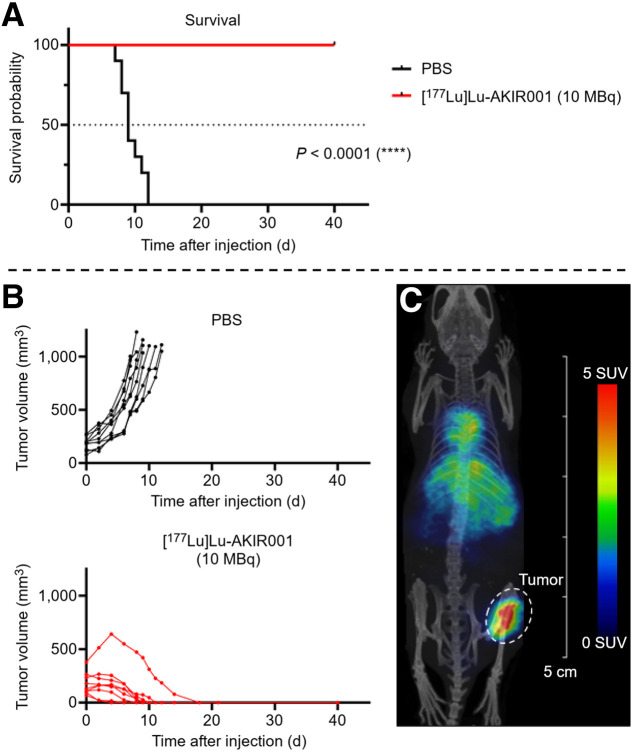
Efficacy studies on BHT-101. (A) Kaplan–Meier survival plot of mice carrying BHT-101 xenografts treated with either placebo (phosphate-buffered saline, *n* = 10) or 10 MBq of [^177^Lu]Lu-AKIR001 (*n* = 9, *N* = 19). (B) Individual tumor growth by treatment group. (C) Small-animal SPECT/CT of mouse carrying BHT-101 xenograft injected with 10 MBq of [^177^Lu]Lu-AKIR001 at 4 d after injection. Tumor-bearing limit was 1,000 mm^3^, and study was concluded on day 40. *P* < 0.0001 was calculated using Mantel–Cox log-rank test comparing placebo control with [^177^Lu]Lu-AKIR001 treatment. Note, the mouse with regrowth in the fractionation group did not reach tumor-bearing limit by the end of the study (day 90).

To evaluate [^177^Lu]Lu-AKIR001 against lower-expressing tumors, efficacy was evaluated in BHT-101 xenografts with moderate CD44v6 expression levels. Placebo mice reached the tumor-bearing limit rapidly (median survival, 9 d), whereas all [^177^Lu]Lu-AKIR001–treated mice achieved 100% complete response with no signs of toxicity, resulting in an undefined median survival (Fig. 4A). One treated mouse with a large initial tumor demonstrated complete regression by day 14, and none of the treated mice had tumor regrowth during the 40-d assessment, nor did the treatment influence animal weight (Fig. 4B; Supplemental Fig. 3C). SPECT imaging confirmed high tumor uptake in BHT-101 xenografts at 4 d after injection (Fig. 4C).

#### Confirmation of Low Uptake in Normal Tissues Through Rabbit Biodistribution Using ^111^In-AKIR001

**FIGURE 5.**
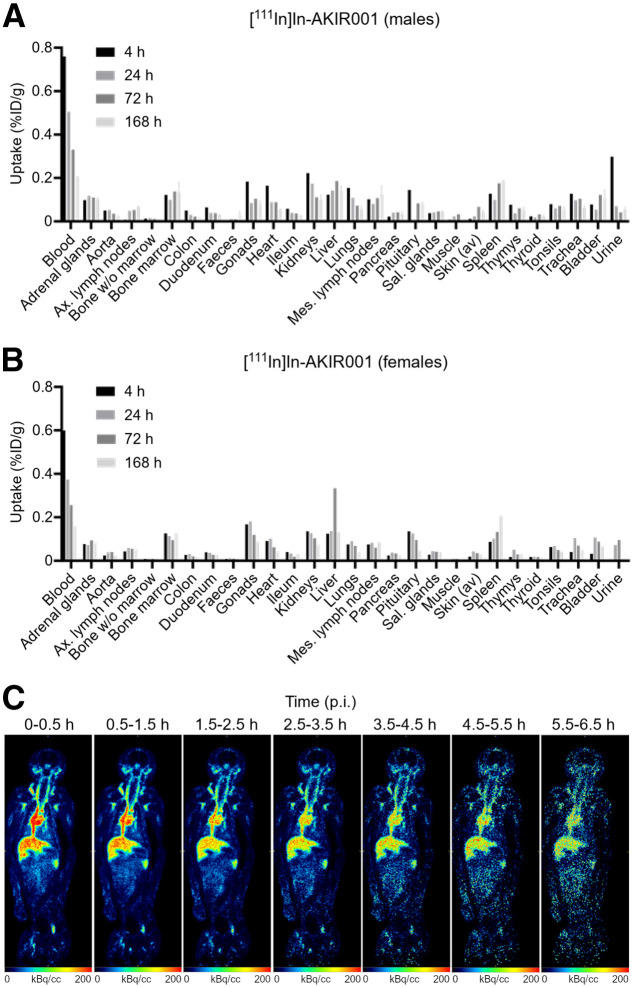
Biodistribution of [^111^In]In-AKIR001 in male (A) and female (B) New Zealand white rabbits (presented as percentage injected dose [%ID] per gram) at 4, 24, 72, and 168 h after injection (*N* = 9; *n* = 5 males and *n* = 4 females). (C) Whole-body ^68^Ga PET images of cynomolgus macaque showing biodistribution of [^68^Ga]Ga-UU-40_IAHA_ at different time points. Images are shown as maximum-intensity projection, with PET in red–green–blue color scale and CT in gray scale. p.i. = after injection.

AKIR001’s cross-reactivity with rabbit CD44v6 enabled biodistribution studies on New Zealand white rabbits using [^111^In]In-AKIR001 (>90% labeling yield) (10). Results demonstrated low uptake in healthy tissues, with most organs peaking at 4 h after injection, except for the liver and spleen, which peaked at later time points (Figs. 5A and 5B).

#### Good-Laboratory-Practice Toxicology Study on Rabbits

A good-laboratory-practice toxicology study on rabbits assessed the potential toxicity of AKIR001 hIgG1 LALA antibody, using nonradiolabeled AKIR001 at more than 20-fold the estimated patient dose. A single intravenous dose of 40 mg/kg caused no notable effects on clinical health, body weight, or behavior. Extensive histologic and clinical pathology evaluations, including hematology, coagulation, and serum chemistry, revealed no significant changes, indicating that even excessive AKIR001 doses were well tolerated in rabbits.

#### Cynomolgus PET/CT

The cross-reactivity of AKIR001 and its parental antibody, UU-40, with cynomolgus CD44v6 was confirmed previously (10). To assess potential off-target binding in normal tissues, a [^68^Ga]Ga-labeled UU-40 imaging study was conducted on a cynomolgus monkey. The antibody was engineered with IAHA mutations to reduce FcRn binding, enabling faster clearance and optimal pharmacokinetics for imaging (15). The [^68^Ga]Ga-UU-40_IAHA_ had a 70% labeling yield and 96% radiochemical purity. Activity was mainly in blood-rich organs, and despite CD44v6 expression in keratinocytes, low uptake was observed in skin model regions such as the earlobe and femoral tissue.

#### Dosimetry

Preclinical dosimetry from A431 xenograft data demonstrated a favorable tumor–to–red marrow dose ratio of 92–153, accounting for tumor growth dilution. Extrapolated to humans via allometric scaling, the estimated tumor-absorbed dose is 11 Gy/GBq, which is 18–150 times higher than absorbed doses to other organs, with tumor–to–red marrow ratios of 35–65, indicating a strong therapeutic window for [^177^Lu]Lu-AKIR001 (Table 2).

**TABLE 2.** ** **Preclinical Dosimetry and Corresponding Extrapolated Human-Absorbed Doses of [^177^Lu]Lu-AKIR001

	**Preclinical tumor-absorbed dose**		**Extrapolated human-absorbed doses**	
**Tissue**	**Absorbed dose (Gy/MBq)**	**Tumor-to-tissue dose ratio**	**Absorbed dose (Gy/GBq)**	**Tumor-to-tissue dose ratio**
RM	0.19	153	0.17	65
Blood-based RM	0.31	92	0.32	35
Tumor	29	1	11	1
Liver	0.36	81	0.29	39
Kidney	0.63	46	0.54	20
Spleen	0.35	82	0.27	41
Lungs	0.44	66	0.60	18
GI	0.12	232	0.14	80
Skin	0.37	79	0.33	34
Muscle	0.11	272	0.07	152
Femur	0.14	210	0.15	72
Carcass	0.30	97	0.23	48

RM = red marrow; GI = gastrointestinal tract.

Data are based on biodistribution data presented in Figure 1A and Table 1 and were adjusted for dilution of activity during A431 xenograft growth.

## DISCUSSION

The results of this study indicate that [^177^Lu]Lu-AKIR001 is a promising candidate for RPT of CD44v6-expressing tumors. This radioantibody–drug conjugate demonstrates high specificity for its target, favorable dosimetry and safety profiles, and potent therapeutic efficacy in preclinical models, strongly supporting its clinical translation.

Traditional RPT approaches have relied on peptide-based agents, which are advantageous for their rapid tumor penetration and fast clearance from circulation. However, for targets such as CD44v6, which possesses unstructured extracellular domains, it is difficult to develop peptides with sufficient affinity and specificity. Radioantibody–drug conjugates such as [^177^Lu]Lu-AKIR001 overcome these limitations by offering higher affinity and prolonged tumor retention, making them especially suitable for RPT with long-lived radionuclides such as ^177^Lu. The success of radioantibody–drug conjugates has already been illustrated with agents such as [^177^Lu]Lu-J591 for PSMA-positive prostate cancer. Notably, CD44v6 is expressed at levels approximately 10 times higher than PSMA in tumors, suggesting that [^177^Lu]Lu-AKIR001 could achieve an even greater therapeutic impact. Furthermore, CD44v6 is broadly expressed across multiple epithelium-derived cancers, positioning [^177^Lu]Lu-AKIR001 as a potential pan-cancer RPT agent. This could significantly expand the clinical use of RPT beyond currently approved agents such as [^177^Lu]Lu-DOTATATE and [^177^Lu]Lu-PSMA-617, which are limited to specific patient populations.

The advantage of targeting highly expressed antigens was demonstrated in biodistribution studies. In A431 xenografts, which express an estimated 3 million antigens per cell, [^177^Lu]Lu-AKIR001 achieved high and sustained tumor uptake (peak > 60 %ID/g), resulting in favorable dosimetry with high tumor-to-organ dose ratios. The peak tumor uptake of [^177^Lu]Lu-AKIR001 was more than 4-fold greater than in published preclinical studies using [^186^Re]Re-BIWA-4, possibly reflecting the higher affinity of AKIR001 (16). This selective accumulation in tumors while limiting systemic exposure is a key feature for maximizing efficacy and minimizing toxicity. In comparison, PSMA-targeting agents typically achieve peak uptakes of about 20 %ID/g in preclinical models (17). The superior uptake of AKIR001 is likely due to both its high affinity and the greater abundance of CD44v6. In addition, the lack of histopathologic findings in the good-laboratory-practice toxicology study on rabbits further support clinical translation of AKIR001, although tissue-specific expression levels likely differ between species. Low uptake in normal tissues was confirmed in rabbit biodistribution and nonhuman primate studies, indicating a favorable dosimetry and safety profile. In the nonhuman primate study, a modified variant of AKIR001 with accelerated plasma clearance was used to allow for high-contrast imaging within the limited sedation window of the animal. Given the high similarity between UU-40 and AKIR001 in binding moiety, and the identical target binding site on CD44v6, this strategy was believed to deliver the best information on normal-tissue distribution in this experimental setting.

A critical aspect of therapeutic development is optimizing dosing to maximize efficacy while limiting toxicity. In our studies, single high doses of [^177^Lu]Lu-AKIR001 achieved high cure rates in A431 and ACT-1 xenografts. To our knowledge, curative doses were not reached with comparable activity doses in preclinical studies with [^186^Re]Re-BIWA-4. Furthermore, fractionated dosing (7.5 MBq × 2) indicated a slight improvement in complete response rates compared with a single, high dose (15 MBq), without increasing hematologic toxicity in the A431 study, suggesting that fractionation could enhance the therapeutic index. Additionally, combining RPT with chemotherapy (CDDP) produced a 25% complete response rate without added toxicity, indicating that combination regimens could further improve patient outcomes. Further, a 100% response rate was achieved in the BHT-101 model with moderate CD44v6 expression levels, indicating that [^177^Lu]Lu-AKIR001 may be efficacious in tumors with lower expression levels. Combined, these efficacy results indicate that [^177^Lu]Lu-AKIR001 is a potent, highly specific, and promising RPT in CD44v6-positive xenografts and surpasses the preclinical data compiled on [^186^Re]Re-BIWA-4.

In summary, [^177^Lu]Lu-AKIR001 demonstrates high specificity, favorable safety and dosimetry, and strong efficacy in preclinical models, supporting its continued clinical development as a potentially transformative RPT agent for a wide range of CD44v6-positive cancers.

## CONCLUSION

[^177^Lu]Lu-AKIR001 is a CD44v6-targeted RPT with strong preclinical efficacy, specificity, and safety. Its broad tumor applicability and its potential for use in combination support clinical evaluation, and it is currently being assessed in a phase 1 trial (NCT06639191) for safety and therapeutic potential in CD44v6-expressing cancers.

## DISCLOSURE

Anja Mortensen, Fredrik Frejd, and Marika Nestor are cofounders of Akiram Therapeutics AB, which owns the intellectual property of the AKIR001 antibody. Peter Bernhardt serves as a consultant for ITM, Affibody AB, and Akiram Therapeutics AB and is the cofounder of Theravison AB. This work was supported by the Swedish Cancer Society (Cancerfonden) (CAN 2,43,485 Pj, 2,22,391 S, 2,43,445 Pj, and 21 0319 FE); Vinnova (2019-01525); the Swedish Research Council (Vetenskapsrådet) (2024-03447); the Sjöberg Foundation (2023-704, 2023-630); the Erling Persson Foundation, (2023-0120); the Swedish Childhood Cancer Fund (Barncancerfonden) (PR2023-0033); the Jubilee Clinic Cancer Research Foundation (2022:412), and the Swedish federal government (ALFGBG-10067793). No other potential conflict of interest relevant to this article was reported.

## Notes

KEY POINTS **QUESTION:** What makes [^177^Lu]Lu-AKIR001 a promising candidate for RPT, what type of preclinical studies and data are needed to complete clinical translation of an RPT, and how does [^177^Lu]Lu-AKIR001 compare with current RPTs? **PERTINENT FINDINGS:** Preclinical results show high tumor specificity, potent efficacy, and minimal off-target toxicity across several tumor models and animal species. Tumor specificity, efficacy and dosimetry demonstrated the feasibility and promise of [^177^Lu]Lu-AKIR001. Safety studies in a secondary animal species were essential to complete and finalize translation. The target, CD44v6, is highly overexpressed in a plethora of cancers and therefore not as limited in its future clinical use as current RPTs. Further, the data presented in this study demonstrate that expression levels are possibly in the millions of antigens per cell, which may be 10-fold greater than expression of other RPT targets such as PSMA. **IMPLICATIONS FOR PATIENT CARE:** In the future, [^177^Lu]Lu-AKIR001 has the potential to offer a precise treatment option for patients with CD44v6-positive solid tumors. By thorough evaluation in a preclinical setting, the risks of potential toxicities in a clinical setting decrease substantially. RPT relies on overexpression of targets on cancer cells for efficacy, and because of the high expression levels of CD44v6, it is possible that [^177^Lu]Lu-AKIR001 will accumulate greater levels of activity in tumors than do current RPTs.
